# Cardiovascular risk factors and cognitive performance in aging

**DOI:** 10.1590/1980-57642016dn11-040015

**Published:** 2017

**Authors:** Juliana Rumy Tsuchihashi Takeda, Tatiane Martins Matos, Juliana Nery de Souza-Talarico

**Affiliations:** 1 RN, Heart Institute (InCor), Faculty of Medicine, University of São Paulo, SP, Brazil.; 2 Master degree, Graduate Program in Adult Healthcare Nursing, School of Nursing, University of São Paulo, SP, Brazil.; 3 PhD, Department of Medical-Surgical Nursing, School of Nursing, University of São Paulo, SP, Brazil.

**Keywords:** memory, cognitive performance, cardiovascular risk factor, preventive health, memória, desempenho cognitivo, fatores de risco cardiovascular, saúde preventiva

## Abstract

**Background.:**

Atherosclerosis in cerebral blood vessels, especially those which compose the Circle of Willis, can lead to reduced supply of oxygen and nutrients to different cortical structures, affecting cognitive function.

**Objective:**

To analyze whether cardiovascular risk factors negatively influence cognitive performance in adults and elderly.

**Methods:**

One hundred twenty-nine participants of both sexes, aged over 50 years, without cognitive or functional impairment were included. Body mass index (BMI), hypertension (HTN), diabetes mellitus (DM), smoking history, plasma levels of total cholesterol, low density lipoproteins (LDL), high density lipoproteins (HDL) and very low density lipoproteins (VLDL) cholesterol, triglycerides, and glucose were the cardiovascular risk factors analyzed. Cognitive assessment was performed using tests of attention, working memory, category fluency and declarative memory.

**Results:**

Controlling for age and education, multivariate linear regression models revealed that higher concentrations of triglycerides, as well as total, LDL and VLDL cholesterol, were associated with poorer performance on the digit span and category fluency tests. Higher HDL concentrations were associated with higher scores on category fluency tasks. Furthermore, higher BMI was associated with poorer delayed recall performance.

**Conclusion:**

The findings revealed that cardiovascular risk factors may negatively impact cognitive performance in aging.

## RESUMO.

**Introdução:**

A aterosclerose nos vasos sanguíneos cerebrais, especialmente naqueles que compõem o Círculo de Willis, pode levar à redução da oferta de oxigênio e nutrientes para diferentes estruturas corticais, afetando a função cognitiva.

**Objetivo:**

Analisar se fatores de risco cardiovascular influenciam negativamente o desempenho cognitivo em adultos e idosos.

**Métodos:**

Foram incluídos cento e vinte e nove participantes de ambos os sexos, com idade superior a 50 anos, sem comprometimento cognitivo ou funcional. Índice de massa corporal (IMC), hipertensão arterial (HAS), diabetes mellitus (DM), história de tabagismo, níveis plasmáticos de colesterol total e lipoproteínas de baixa densidade (LDL), lipoproteínas de alta densidade (HDL) e lipoproteínas de baixa densidade (VLDL), triglicerídeos e glicemia foram os fatores de risco cardiovascular analisados. A avaliação cognitiva foi realizada utilizando testes de atenção, memória operacional, fluência verbal e memória declarativa.

**Resultados:**

Controlando para idade e escolaridade, os modelos de regressão linear multivariada revelaram que concentrações mais elevadas de triglicerídeos, bem como colesterol total, LDL e VLDL, estão associadas com pior desempenho nos testes de extensão de dígitos e fluência verbal. Além disso, concentrações maiores de HDL se correlacionaram com pontuações maiores nas tarefas de fluência verbal. IMC maior se associou com menor desempenho na evocação tardia.

**Conclusão:**

Os achados revelaram que fatores de risco cardiovascular podem afetar negativamente o desempenho cognitivo durante o envelhecimento.

## INTRODUCTION

Complaints of memory decline during aging have been the subject of many studies, in an attempt to identify factors that might explain the cognitive variability seen among adults and the elderly. Many elders have cognitive performance that is equal to or even better than young adults; although common, memory decline cannot be considered part of natural aging.^1^^-^ 6

Genetic, epigenetic and environmental factors have been considered in research investigating how healthy habits and lifestyle can influence brain function and human behavior throughout life.^4^

Most available evidence describes the effect of cardiovascular risk on cognitive performance in individuals who already have some degree of cognitive impairment.^7^ However, it is still unclear whether these factors also negatively affect cognitive performance in healthy adults and elderly. This is particularly relevant, since actions for prevention and control of cardiovascular risk factors can reduce not only the risk for ischemic cardiomyopathies, but can also promote wellbeing and quality of life during aging.

Anatomical and functional age-related changes allied with exposure to cardiovascular risk factors may compromise cerebral blood flow which may in turn negatively influence cognitive performance.^3^^,^ 5

Changes in cardiac output, increase in cholesterol levels and peripheral vascular resistance, systemic blood pressure, body mass index (BMI), and body fat number among the cardiovascular risk factors observed during aging which may influence memory decline.^6^

Atherosclerosis in cerebral blood vessels, especially those that comprise the Circle of Willis, can lead to a reduced supply of oxygen and nutrients to different cortical structures, affecting cognitive function in patients with cardiovascular diseases.^8^ The very high blood pressure can interfere with microcirculation and cause cerebral ischemia.^2^^,^ 8

The control of cardiovascular risk factors, such as hypertension, diabetes and dyslipidemia, is associated with better cognitive performance.^2^^,^ 8 Additionally, post-mortem analysis has revealed a positive association between high cholesterol and amyloid deposits, one of the most important biomarkers of Alzheimer's disease (AD).^9^ Similarly, previous studies have showed that cardiovascular risk factors are associated with poor memory performance and executive function in midlife adults from developed countries.^10^^,^ 11 However, this is poorly understood among individuals from low- and middle-income countries, where socioeconomic inequality may limit access to quality health services and thus, the control of cardiovascular risk factors.

This study aimed to examine whether modifiable cardiovascular risk factors negatively influence cognitive performance in Brazilian adults and older adults.

## METHODS

### Ethical procedures.

All participants were volunteers and signed the consent form for the study, approved by the Research Ethics Committee of the Federal University of São Paulo - UNIFESP (n. 0823/09).

### Participants.

One hundred twenty-nine elderly individuals were included, with preserved, intact cognitive function; 83.6% (107 individuals) were female, with mean age of 65.5 (SD ± 8.0) years, and mean education of 9.78 (SD ± 4.45) years of study.

Eligibility criteria for the study participants were: aged over 50 years, both sexes, literate, with education ≥ four years; intact cognitive and functional features preserved (according to performance evaluation on the Mini-Mental State Examination (MMSE) and the Informant Questionnaire on Cognitive Decline in the Elderly (IQCODE) for dementia detection). Individuals were excluded according to the following criteria: diagnosis of neurological, neurodegenerative and/or psychiatric disease; use of psychoactive drugs; a history of alcohol or drug abuse in the past year, or previously for a long period; smokers or ex-smokers for less than ten years.

The risk factors established by the American Heart Association^12^ include BMI, hypertension (HTN), diabetes mellitus (DM), smoking history, blood glucose, triglycerides, total cholesterol and LDL, HDL and VLDL cholesterol. Blood samples were analyzed by the *Associação Fundo de Incentivo à Pesquisa* (AFIP).

### Cognitive assessment.

The cognitive abilities evaluated were: attention, working memory, category fluency, and short and long-term declarative memory.

Attention and working memory performance were evaluated using the Forward Digit Span (FDS) and Forward Corsi Blocks (FCB) tests which evaluate attention, and the Backward Digit Span (BDS) and Backward Corsi Blocks (BCB) tests that evaluate working memory. In the FDS, a sequence of digits was read out at a rate of one number per second. After reading, the individual is asked to repeat the numbers in the order they were read out. If errors occur, the individual can retake the test with a sequence of the same number of digits. In the BDS, the individual must remember the sequence backwards. The test assesses the storage capacity and reverberation in immediate verbal memory (phonological loop), and the ability to maintain and manipulate information (central executive). The scores for both tests were the maximum number of digits the individuals repeated in sequence correctly, which ranged from three to six digits.

In the Corsi blocks test, sets of nine blocks were presented (black boxes on white backgrounds) arranged randomly on a computer screen. In the first sequence, two blocks changed colors sequentially at one-second interval, and the individual had to indicate that block change sequence. When answering correctly, the length of the sequence was increased until the participant committed two errors in the same sequence. For the indirect order (BCB), the individual had to indicate the sequence backwards. The scores for both tests were the maximum number of digits or blocks correctly repeated in the sequence, which ranged from three to six digits or blocks.

For assessment of category fluency, the FAS version was used, in which the participant had to state as many words as possible beginning with the letters F, A, and S, with one minute for each letter. The objective of this test is to evaluate spontaneity in generating words. It constitutes an executive function test, because the participant must apply a set of rules with which he/she must comply during the generation of words. The score corresponds to the number of words stated for each letter, and mistakes (for example, repeated words or those that did not begin with the proposed letter). Category fluency is also evaluated, using animals, fruits and musical instruments stated in one minute.

Declarative memory was assessed using the California Verbal Learning Test (CVLT). The test measures the verbal learning process, and the amount of material acquired and retained using a verbal memory task. A list containing 16 items was presented, classified into categories of clothing, vegetables, tools, and fruit. The individual had five attempts to recall the items and remember the list immediately after its presentation. Thirty minutes after the immediate recall list (learning), the participant had to recall the items again, without being reread the list (delayed recall). The score is the number of items recalled during the five attempts, that is, a point is given for each correct item.

Data collection procedures. Initially the study was publicized in the media, through the radio, newspaper and internet, spontaneously attracting volunteers interested in participating in the study. These individuals contacted the telephone survey team to express their interest in participating. During this telephone contact, the study candidate answered questions relevant to inclusion and exclusion criteria. Those who were included in the study, in accordance with the criteria, were scheduled for individual interviews at the Department of Psychobiology of UNIFESP. In this interview, participants were assessed for functional and cognitive performance, and those with incompatible performance for age and level of education, and with pre-established scores on the MMSE and IQCODE, were excluded.^13^^,^ 14

Informed consent was obtained from eligible participants. Blood samples were collected for laboratory analysis, and then sociodemographic and cognitive assessment questionnaires were immediately administered. Anthropometric measurements (weight and height) were obtained using properly calibrated manual scales.

### Statistical analyses.

Data were analyzed descriptively using means, standard deviation (SD) and frequency (absolute and relative). For analyses of the learning curve in the CVLT test, the analysis of variance (ANOVA) for repeated measures was used with the Greenhouse correction method in the absence of sphericity. To investigate the association between cognitive assessment test scores and cardiovascular risk factors, multivariate linear regression analysis was performed with age and education as potential confounders. The Statistical Package for the Social Sciences (SPSS), version 14.0 was used. The significance level was set at p < 0.05.

## RESULTS

Cardiovascular risk factor profiles and cognitive assessment. More than half the sample (58.6%) had HTN, 23.8% had stopped smoking for at least 10 years, and 8.7% had type 2 DM. About 40% of the sample was overweight or obese, according to the BMI cutoff value > 25 for individuals up to 59 years, and BMI > 27 for elderly aged 60 or older.^15^ Regarding medication, 59.7% (n = 77) used antihypertensive drugs, statins, oral hypoglycemic agents and antidiuretics. The lipid profile and mean glucose concentrations are shown in Table 1.

The participants had cognitive and functional performance considered to be within normal range.^13^^,^ 14 Table 1 shows the cognitive performance of participants.

**Table 1:** Sample characteristics according to cognitive and functional performance and cardiovascular risk factors.

Variables		Minimum	Maximum	Mean	± SD
**Cardiovascular risk factors**	**Glucose (mg/dL)**	77	207	99.8	17.6
	• Total cholesterol (mg/dL)	123	315	195.3	37.0
	• High density lipoprotein (mg/dL)	30	105	60.3	15.2
	• Low density lipoprotein (mg/dL)	32	230	113.4	31.0
	• Very low density lipoprotein (mg/dL)	9	73	22.0	10.5
	• Triglycerides (mg/dL)	44	364	109.9	52.6
	• Body Mass Index (kg/m^2^)	15.82	39.26	25.99	4.27
**Cognitive performance**	**Global**				
	• MMSE^a^	24	30	27.6	1.5
	**Attention**				
	• Forward Digit Span	2	6	4.5	0.9
	• Forward Corsi Blocks	1	6	3.2	1.4
	**Working memory**				
	• Backward Digit Span	1	6	2.7	1.2
	• Backward Corsi Blocks	1	6	3.3	1.3
**Verbal fluency**	F	1	21	11.6	3.9
	A	2	19	10.4	3.6
	S	2	19	10.5	3.6
**Cognitive decline**	IQCODE^b^	1.23	3.3	2.8	0.7

a Mini-Mental State Exam;

b Informant Questionnaire on Cognitive Decline in the Elderly.

**Figure 1:**
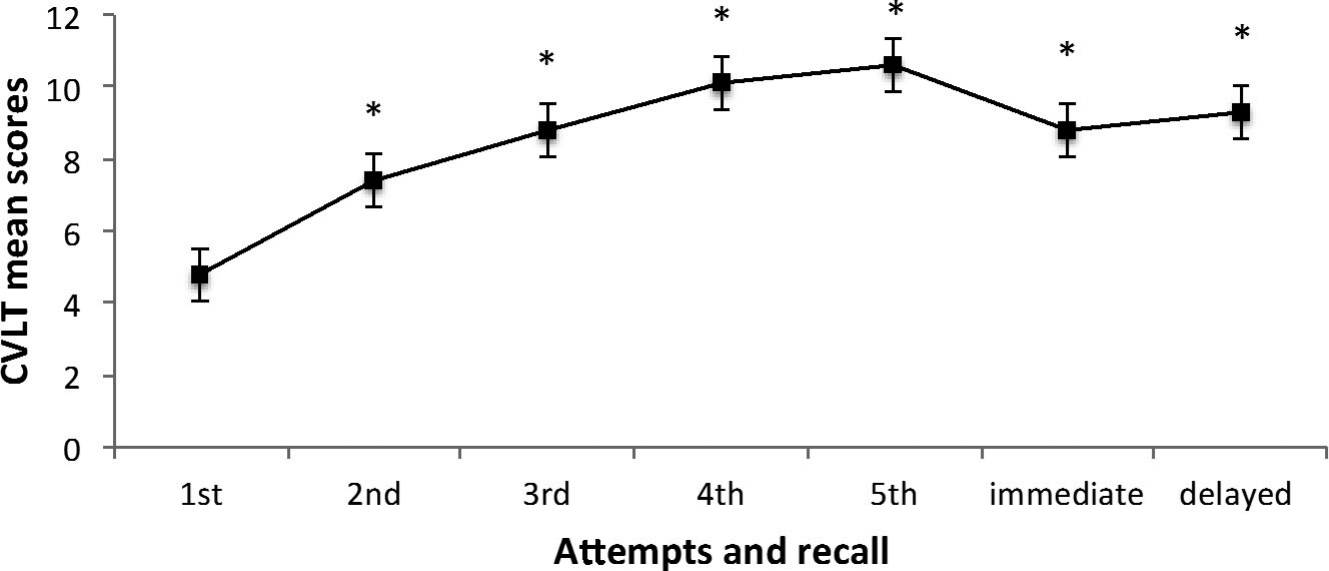
Means of immediate short-term and delayed recall of California Verbal Learning Test. * Indicates significant difference between the first and subsequent recalls (p <0.05). The bars represent standard error.

Regarding the CVLT test, an effect of time on immediate short- and long-term recall of the list items was observed (F (762.685) = 172.9, p < 0.001; Figure 1), evidencing a learning effect after five attempts to memorize the list.

Association between cardiovascular risk factors and cognitive performance. Controlling for age and education, multivariate linear regression models revealed that higher concentrations of triglycerides, as well as total, LDL and VLDL cholesterol, were associated with poorer performance on the digit span and category fluency tests. Higher HDL concentrations were associated with higher scores on category fluency tasks. Furthermore, higher BMI was associated with poorer delayed recall performance. No association was observed between cognitive performance and glucose, HTN, DM or previous smoking history (Table 2).

**Table 2:** Multivariate linear regression coefficients between cardiovascular risk factors and cognitive test scores.

									CVLT^e^			
	FDS^a^		BDS^b^		FCB^c^		BCB^d^		Short-term recall		Delayed recall	
	B (p)		B (p)		B (p)		B (p)		B (p)		B (p)	
Age	–0.026 (0.012)*		–0.006 (0.705)		0.002 (0.913)		–0.002 (0.929)		–0.103 (0.010)*		–0.144 (< 0.001)*	
Education	0.021 (0.287)		0.082 (0.002)*		0.095 (0.003)*		0.070 (0.016)*		0.026 (0.722)		0.061 (0.400)	
Glucose	0.002 (0.633)		–0.006 (0.325)		–0.005 (0.514)		0.003 (0.663)		–0.001 (0.960)		0.009 (0.599)	
Total cholesterol	–0.058 (0.705)		–0.109 (0.615)		0.252 (0.343)		0.225 (0.357)		1.027 (0.075)		0.896 (0.125)	
HDL cholesterol	0.058 (0.705)		0.106 (0.627)		–0.243 (0.359)		–0.215 (0.380)		–1.044 (0.071)		–0.027 (0.058)	
LDL cholesterol	0.057 (0.711)		0.103 (0.636)		–0.260 (0.327)		–0.231 (0.345)		–1.018 (0.077)		–0.898 (0.124)	
VLDL cholesterol	–0.021 (0.029)*		–0.028 (0.038)*		0.229 (0.400)		0.190 (0.451)		1.061 (0.074)		0.893 (0.137)	
Triglycerides	–0.008 (0.003)*		–0.008 (0.031)*		–0.095 (0.375)		–0.082 (0.410)		–0.417 (0.075)		–0.350 (0.138)	
BMI^f^	0.003 (0.896)		0.053 (0.082)		–0.033 (0.364)		–0.024 (0.487)		0.011 (0.887)		–0.188 (0.033)*	
Smoking history	–0.137 (0.662)		0.009 (0.891)		–0.014 (0.968)		–0.225 (0.807)		1.035 (0.209)		0.367 (0.659)	
Diabetes mellitus	–0.086 (0.852)		–0.072 (0.895)		–0.367 (0.476)		–0.473 (0.726)		–0.157 (0.896)		–0.505 (0.679)	
Hypertension	–0.062 (0.809)		0.046 (0.880)		0.184 (0.521)		–1.230 (0.107)		–0.827 (0.219)		–0.535 (0.432)	
	R^2^ 0.139	p 0.002	R^2^ 0.128	p 0.005	R^2^ 0.089	p 0.003	R^2^ 0.059	p 0.016	R^2^ 0.117	p 0.071	R^2^ 0.153	p < 0.001
	**Phonemic category fluency**						**Semantic category fluency**					
	**F**		**A**		**S**		**Animals**		**Fruits**		**Instruments**	
	**B (p)**		**B (p)**		**B (p)**		**B (p)**		**B (p)**		**B (p)**	
Age	–0.017 (0.715)		0.026 (0.526)		0.036 (0.415)		–0.064 (0.214)		–0.073 (0.040)*		–0.020 (0.604)	
Education	0.371 (< 0.001)*		0.351 (< 0.001)*		0.322 (<0.001)*		0.399 (< 0.001)*		0.085 (0.191)		0.227 (0.001)*	
Glucose	–0.004 (0.819)		–0.001 (0.945)		0.005 (0.779)		–0.012 (0.580)		–0.017 (0.279)		0.003 (0.832)	
Total cholesterol	0.246 (0.705)		–0.369 (0.523)		–0.076 (0.903)		0.730 (0.307)		–0.104 (0.025)*		0.644 (0.235)	
HDL cholesterol	–0.234 (0.719)		0.388 (0.502)		0.096 (0.876)		–0.740 (0.301)		0.085 (0.017)*		–0.664 (0.221)	
LDL cholesterol	–0.251 (0.699)		0.349 (0.545)		0.063 (0.919)		–0.725 (0.310)		–0.115 (0.020)*		–0.647 (0.232)	
VLDL cholesterol	0.204 (0.760)		–0.445 (0.454)		–0.055 (0.014)*		0.726 (0.324)		0.124 (0.814)		0.658 (0.238)	
Triglycerides	–0.097 (0.712)		0.158 (0.499)		0.045 (0.857)		–0.294 (0.309)		–0.021 (0.025)*		–0.255 (0.244)	
BMIg	0.152 (0.091)		0.089 (0.261)		0.053 (0.542)		–0.227 (0.021)*		0.770 (0.275)		–0.188 (0.009)*	
Smoking history	0.225 (0.827)		0.556 (0.496)		1.293 (0.148)		–0.703 (0.480)		–0.417 (0.525)		0.272 (0.710)	
Diabetes mellitus	–1.650 (0.706)		–1.650 (0.171)		–0.508 (0.697)		–1.517 (0.300)		0.101 (0.964)		0.609 (0.571)	
Hypertension	–0.148 (0.104)		–0.148 (0.825)		–0.512 (0.481)		0.130 (0.873)		–0.812 (0.173)		–1.021 (0.090)	
	R^2^ 0.204	p < 0.001	R^2^ 0.210	p < 0.001	R^2^ 0.192	p < 0.001	R^2^ 0.207	P < 0.001	R^2^ 0.098	p 0.083	R^2^ 0.170	P 0.027

a Forward Digit Span;

b Backward Digit Span;

c Forward Corsi Block;

d Backward Corsi Block;

e California Verbal Learning Test;

f Body Mass Index.

* indicates significant association (p < 0.05).

## DISCUSSION

This study allowed us to ascertain that cardiovascular factors are associated with low cognitive performance. Individuals with higher levels of LDL, VLDL, triglycerides and BMI showed poorer performance in attention, working memory, category fluency and delayed recall, suggesting a negative influence of lipids on memory during aging.

Corroborating our findings, previous studies show increased LDL in patients with dementia or in those who have suffered a stroke.^7^ Furthermore, changes in serum lipids and lipoproteins are associated with the development of cerebrovascular disease.^7^ Accordingly, adults with high HDL had better performance on language tests,^16^ immediate recall and the MMSE,^17^ reinforcing that the bad cholesterol fraction can negatively affect memory performance in older adults. However, findings obtained in cognitively healthy, nonagenarian and centenarian elderly revealed no significant association between dyslipidemia and cognitive impairment.^18^^-^ 20 Also, treatment with lipid-lowering agents did not result in improvement in memory performance or language.^18^

In addition, we found that the greater the BMI, the poorer the category fluency and delayed recall performance. Given the category fluency test evaluates not only executive function, but also the ability to generate words, it is reasonable to assume that the negative correlation between BMI and category fluency may be related to a slowing in information processing speed. Supporting this interpretation, some authors found a significant association between high BMI and worse overall cognitive performance,^21^ memory decline,^22^ poor attention and processing speed.^23^ Although these authors attributed age to slower processing speed, in the current study, BMI was negatively associated with category fluency even after controlling for age and education level.

Although the mechanisms by which cardiovascular risk factors may compromise cognitive performance during aging are unclear, some hypotheses have been discussed. High concentration of LDL, as an independent predictor of coronary artery disease and carotid artery atherosclerosis,^2^^,^ 7 can lead to embolism and cerebral hypoperfusion and subsequent cognitive decline. Hypertension may induce atherosclerosis and cause capillary damage, leading to hypoxia, cerebral ischemia, and clinical and subclinical brain damage.^24^ Obesity can reduce cardiovascular capacity, promote inflammation, and lead to endocrine disruption, thereby influencing cognitive performance.^21^ Furthermore, a high BMI is associated with decreased white matter in the brain when weight loss is induced by dieting.^23^

It is noteworthy that the associations observed in this study were obtained with mean cholesterol and triglyceride levels within the normal range, and only a few individuals actually had dyslipidemia. This suggests that lipid indicators such as cholesterol and triglycerides do not necessarily need to be overly high to negatively influence cognitive performance. Thus, it is possible that cognitive performance is modulated by lipid indicators in an inverted U-shape function, in which optimal lipid concentrations are associated with improved memory performance, whereas overly high or low concentrations can produce cognitive decline. Supporting this interpretation, some studies have found a positive correlation between cholesterol, BMI and cognitive performance.^25^ It should be noted that these lipids are natural components of the body and necessary for the formation of neuronal myelin sheath, and hence for the conduction of nerve impulses.

It is important to consider that the sample analyzed in this study was composed of predominantly female individuals over 60 years of age, which raises the possibility of selection bias in the results. However, we emphasize that the variable “age” was included as a covariate in the statistical analysis. In addition, non-random selection of participants can compromise the external validity of the results, since it may not reflect the actual health scenario of the adult and elderly populations. In this sense, the results may have been underestimated and the observed correlations might be stronger, if we consider that many older people do not spontaneously seek health services to assess their metabolic status and cognitive performance.

In summary, our findings support the hypothesis that cardiovascular risk factors, specifically natural lipids, can negatively impact memory and verbal fluency performance of individuals throughout the aging process. The study provides evidence for health professionals regarding the earlier identification of risk factors that may compromise cognitive performance. Given the relevance of optimal cognitive performance for activities of daily living and successful aging, our findings reinforce the importance of preventive strategies for managing the occurrence of cardiovascular risk factors to reduce the prevalence of ischemic cardiomyopathies, but also suggest that such strategies may have a positive impact on cognitive performance during aging.
